# Outcome of HIV-associated *Pneumocystis *pneumonia in hospitalized patients from 2000 through 2003

**DOI:** 10.1186/1471-2334-8-118

**Published:** 2008-09-16

**Authors:** Saba Radhi, Travis Alexander, Michelle Ukwu, Samer Saleh, Alison Morris

**Affiliations:** 1 Department of Medicine, Division of Pulmonary and Critical Care Medicine and the Will Rogers Institute Pulmonary Research Center, University of Southern California, Los Angeles, CA, USA; 2 Department of Medicine, Division of Pulmonary, Allergy, and Critical Care Medicine, University of Pittsburgh, Pittsburgh, PA, USA

## Abstract

**Background:**

*Pneumocystis *pneumonia (PCP) remains a leading cause of morbidity and mortality in HIV-infected persons. Epidemiology of PCP in the recent era of highly active antiretroviral therapy (HAART) is not well known and the impact of HAART on outcome of PCP has been debated.

**Aim:**

To determine the epidemiology of PCP in HIV-infected patients and examine the impact of HAART on PCP outcome.

**Methods:**

We performed a retrospective cohort study of 262 patients diagnosed with PCP between January 2000 and December 2003 at a county hospital at an academic medical center. Death while in the hospital was the main outcome measure. Multivariate modeling was performed to determine predictors of mortality.

**Results:**

Overall hospital mortality was 11.6%. Mortality in patients requiring intensive care was 29.0%. The need for mechanical ventilation, development of a pneumothorax, and low serum albumin were independent predictors of increased mortality. One hundred and seven patients received HAART before hospitalization and 16 patients were started on HAART while in the hospital. HAART use either before or during hospitalization was not associated with mortality.

**Conclusion:**

Overall hospital mortality and mortality predictors are similar to those reported earlier in the HAART era. PCP diagnoses in HAART users likely represented failing HAART regimens or non-compliance with HAART.

## Background

The introduction of highly active antiretroviral therapy (HAART) led to dramatic declines in morbidity and mortality of HIV-infected patients [1,2]. Although a marked reduction in *Pneumocystis *pneumonia (PCP) was documented in the HAART era, PCP remains the leading AIDS-defining opportunistic infection in the United States [3-6]. Mortality from PCP has changed multiple times during the AIDS epidemic with the latest mortality of hospitalized patients with PCP reported to be 11% in the early HAART era [7]. For patients requiring intensive care, studies performed shortly after the introduction of HAART showed a mortality ranging from 53 to 62% [8-10]. Certain clinical factors such as low serum albumin, need for mechanical ventilation, and development of a pneumothorax were predictive of mortality [9,11-15]. HAART has now been widely available for approximately twelve years. Whether the epidemiology of PCP or predictors of outcome of HIV-infected patients with PCP have changed in the recent HAART era is unknown.

The relationship of HAART use to outcome of PCP is also not well-defined. A previous study found that intensive care unit (ICU) patients receiving HAART during hospitalization had improved mortality [9]. This study was performed early in the HAART era and examined a small number of patients who were admitted to intensive care. A more recent study found that PCP patients admitted to the ICU after mid-1996 had improved outcomes compared to those admitted before the widespread availability of HAART, independent of HAART use [15]. Other studies have not found an association of HAART use and outcome in HIV-infected ICU patients with a variety of diagnoses, but no studies have examined HAART in non-ICU patients with PCP. The exact relationship of HAART and outcome in PCP is unknown and is important for guiding care of these patients.

We studied patients with PCP at Los Angeles County-University of Southern California (LAC-USC) hospital from 2000 to 2003 to determine the epidemiology of PCP and investigate the association of HAART and mortality.

## Methods

### Subjects

Subjects were HIV-infected adults who were discharged from or died in LAC-USC with a diagnosis of PCP between January 1, 2000 and December 31, 2003. We conducted a computerized hospital records search using ICD-9 codes to identify those patients with either a microscopically-confirmed or an empiric diagnosis of PCP. Repeat admissions occurring more than two weeks after hospital discharge were considered as separate admissions. Readmissions within two weeks of discharge were considered as single admissions. The University of Southern California Institutional Review Board approved the study.

### Data collection

Data were collected by standardized chart review using pre-determined definitions and included demographic information, medical history, laboratory data, and medication use. A PCP diagnosis was considered definitive if organisms were visualized on either induced sputum or bronchoscopic samples. A diagnosis was considered empiric if subjects presented with a compatible clinical course, were treated for PCP, and received a discharge diagnosis of PCP. The development of complications including pneumothorax and need for ICU admission or mechanical ventilation (data on non-invasive modes of ventilation were not available) was recorded. HAART was defined as the use of at least three drugs from at least two classes. Details of HAART use included both pre-admission treatment and initiation or continuation of HAART during hospital admission. Number of days a patient received HAART while in the hospital and missed doses were also noted. In-hospital mortality was the primary outcome measure.

### Statistical analysis

Stata 9.0 (College Station, Texas) was used for all analyses, and statistical significance determined for a p-value of ≤ 0.05. Demographic and clinical data were described using mean and standard deviation or median and range depending on normality of the data. Dichotomous variables were described using percentages. Plasma HIV RNA viral level values were log-transformed. Analyses including CD4 cell counts and HIV RNA viral levels were limited to patients who had values available within six months of admission. Clinical characteristics of subjects were compared according to HAART use. Univariate analyses were performed to determine predictors of mortality. For continuous variables, either Wilcoxon rank sum or Student's t-test was used to compare groups. Differences in categorical variables for survivors and non-survivors were assessed using the chi-square test or Fisher's exact and p-values adjusted for multiple comparisons as appropriate. These analyses were repeated for subjects with a definitive diagnosis of PCP and those with an empiric diagnosis. Stepwise forward and backward multivariate logistic regression was performed to determine variables predictive of in-hospital mortality. The robust standard errors method was used to account for repeat admissions. Variables were included in the model if they reached a significance level of p < 0.1 in univariate analysis. Univariate predictors were similar for both definitive and empiric PCP diagnoses; therefore, these groups were combined in multivariate analyses to improve power. Because we were specifically interested in the effect of HAART use, we performed several Cox proportional hazard models adjusting for relevant variables examining differing aspects of HAART use and impact on survival time.

## Results

### Demographics and clinical characteristics

**Figure 1:**
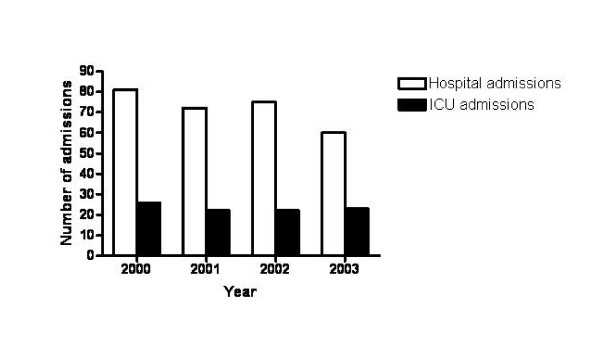
Number of cases of *Pneumocystis *pneumonia admitted to Los Angeles County-University of Southern California hospital and number of cases requiring intensive care unit (ICU) admission by year.

284 HIV-infected adults had 355 admissions for PCP at LAC-USC during the study period. A total of 292 admissions for 262 patients were available for review. Clinical data for the remaining 63 admissions were unavailable due to random loss in medical records. Analyses were repeated including only initial PCP admissions and results were essentially unchanged. Number of patients hospitalized for PCP and number of patients with PCP admitted to the ICU over the course of the study are shown in Figure 1.

Clinical characteristics of subjects are shown in Table 1. Median age of the subjects was 40 years (range 23 to 68). The majority (n = 246, 84.2%) were males, and most subjects were Hispanic/Latino (n = 151, 51.7%). Approximately 60% had a previous diagnosis of AIDS based either on an opportunistic infection or a CD4 cell count below 200 cells/μl. Sixty-eight (27.6%) had a history of pneumonia other than PCP with 19.1% of these subjects reporting previous bacterial pneumonia and 55.9% reporting previous tuberculosis. Almost one quarter (n = 72, 24.7%) had a past history of PCP, and only 31.5% (n = 92) were using PCP prophylaxis. Of the patients not reporting use of PCP prophylaxis, 70 (35.0%) should have been receiving prophylaxis based on a CD4 cell count below 200 cells/μl and known HIV status. The remaining patients either had a CD4 cell count above 200 cells/μl or did not know that they were HIV-infected. Only one patient had an undetectable plasma HIV viral level (less than 50 copies/ml).

**Table 1:** Characteristics of HIV-infected patients with *Pneumocystis *pneumonia according to HAART status on admission.

**Characteristic**	**All (n = 292)**	**Not on HAART (n = 178)**	**On HAART (n = 107)**
**Age, median years, (range)**	40 (23–68)	40 (23–63)	41 (21–68)

**Gender, n (%)**			
Male	246 (84.2)	152 (85.4)	90 (84.1)
Female	46 (15.8)	26 (14.6)	17 (15.9)

**Race/ethnicity, n (%)**			
White	30 (10.3)	17 (9.6)	12 (11.2)
Black	102 (34.9)	63 (35.4)	37 (34.6)
Hispanic/Latino	151 (51.7)	91 (51.1)	57 (53.3)
Other/unknown	9 (3.1)	7 (3.9)	1 (0.9)

**HIV risk factor, n (%)**			
Men who have sex with men	97 (33.2)	58 (32.6)	38 (35.5)
Intravenous drug use	32 (11.0)	18 (10.1)	13 (12.1)
Heterosexual	80 (27.4)	50 (28.1)	29 (27.1)
Blood transfusion	3 (1.0)	1 (0.6)	2 (1.9)
Unknown	80 (27.4)	51 (28.7)	25 (23.4)

**Medical history, n (%)**			
Initial HIV diagnosis	80 (27.4)	79 (44.4)	0 (0)*
History of PCP	72 (24.7)	26 (14.6)	43 (40.2)*
Use of PCP prophylaxis	92 (31.5)	30 (16.9)	60 (56.1)*
Use of HAART	107 (36.6)	0 (0)	107 (100)*
Current smoker	116 (39.7)	74 (41.6)	41 (38.3)

**Laboratory values**			
CD4, cells/μl, median (range)(n = 131)	19 (0–697)	18 (1–267)	22 (0–697)
HIV viral level, log copies/ml, median (range)(n = 57)	5.3 (1.7–5.9)	5.3 (1.7–5.9)	5.3 (2.6–5.9)
Albumin, g/dl, median (range)	2.5 (0.6–4.1)	2.5 (0.6–3.8)	2.4 (0.6–4.1)
LDH, U/l, median (range)	332 (119–1425)	331 (119–1425)	323 (119–962)
Alveolar-arterial oxygen gradient, mm Hg, median (range)(n = 129)	43.3 (18.0–89.0)	44.6 (18.0–89.0)	38.5 (21.8–73.0)

**Complications, n (%)**			
ICU admission	100 (34.2)	66 (37.1)	29 (27.1)
Mechanical ventilation	39 (13.4)	25 (14.0)	11 (10.3)
Pneumothorax	20 (6.8)	17 (9.6)	2 (1.9)^
Respiratory failure after 5 days	10 (3.4)	8 (4.5)	2 (1.9)
Change of PCP therapy	14 (4.8)	10 (5.6)	4 (3.7)

**Outcome**			
Died in-hospital	34 (11.6)	21 (11.8)	10 (9.3)

**Length of stay, median days (range)**	8 (1–89)	9 (1–89)	6 (2–69)*

Abbreviations: HAART, highly active antiretroviral therapy; ICU, intensive care unit; LDH, lactate dehydrogenase; PCP, *Pneumocystis *pneumonia.

Note: HAART use was unavailable for 7 subjects.

*p ≤ 0.001

^p = 0.01

Two hundred and forty-five subjects (83.9%) were initially treated with trimethoprim/sulfamethoxazole (TMP/SMX). Approximately five percent (n = 14) had a change in PCP regimen. Reasons noted for treatment change were development of adverse effects (n = 9, 64.3%) or treatment failure (n = 3, 21.4%). Treatment decisions and dosing regimens were not standardized and were based on physician preference. The majority of subjects (n = 209, 71.6%) received steroids. Of those with arterial blood gas data available, 84.7% of patients meeting clinical criteria received steroids. There was no association of mortality with appropriate steroid use, likely because there were a small number of patients who did not receive steroids when indicated.

One hundred and twenty seven (43.5%) diagnoses were made empirically based on clinical presentation and response to anti-*Pneumocystis *treatment. Patients who had an empiric diagnosis differed from those in whom a definitive diagnosis was pursued (Table 2). Black subjects were more likely to be treated empirically than other groups (odds ratio [OR] = 2.6, 95%CI [confidence interval] = 1.60–4.30, p < 0.001). Empirically-diagnosed patients were more likely to have known HIV on admission (OR = 1.89, 95%CI = 1.10–3.25, p = 0.02), to have a history of PCP (OR = 1.80, 95%CI = 1.03–3.07, p = 0.04), and to be using HAART (OR = 1.8, 95%CI = 1.11–2.92, p = 0.02). Patients who were empirically diagnosed also tended to be less severely ill with less frequent ICU admissions (OR = 0.42, 95%CI = 0.25–0.71, p = 0.001), reduced need for mechanical ventilation (OR = 0.40, 95%CI = 0.19–0.86, p = 0.02), fewer pneumothoraces (OR = 0.13, 95%CI = 0.03–0.57, p = 0.002), and lower mortality (6.3% versus 15.8%, OR = 0.36, 95%CI = 0.16–0.82, p = 0.02). They were also less likely to have PCP therapy changed (OR = 0.09, 95%CI = 0.012–0.72, p = 0.005). In multivariate modeling, being black was the only independent predictor of an empiric diagnosis (OR = 2.4, 95%CI = 1.40–4.13, p = 0.001).

**Table 2:** Characteristics of HIV-infected patients with empiric compared to definitive diagnoses of *Pneumocystis *pneumonia.

**Characteristic**	**Empiric n = 127**	**Definitive n = 165**	**p-value**
**Age, median years, (range)**	41 (23–68)	40 (24–60)	NS

**Gender, n (%)**			NS
Male	107 (84.3)	139 (84.2)	
Female	20 (15.7)	26 (15.8)	

**Race/ethnicity, n (%)**			< 0.001*
White	14 (11.0)	16 (9.9)	
Black	60 (47.2)	42 (26.0)	
Hispanic/Latino	53 (41.7)	98 (60.5)	
Other/unknown	0 (0)	9 (5.5)	

**HIV risk factor, n (%)**			NS
Men who have sex with men	39 (30.7)	58 (35.2)	
Intravenous drug use	18 (14.2)	14 (8.5)	
Heterosexual	36 (28.3)	44 (26.7)	
Blood transfusion	1 (0.8)	2 (1.2)	
Unknown	33 (26.0)	47 (28.5)	

**Medical history, n (%)**			
Initial HIV diagnosis	26 (20.5)	54 (32.7)	0.02
History of PCP	38 (29.9)	34 (20.6)	0.04
Use of PCP prophylaxis	44 (34.6)	48 (29.1)	
Use of HAART	57 (44.9)	50 (30.3)	0.02
Current smoker	54 (42.5)	62 (37.6)	

**Laboratory values**			NS
CD4, cells/μl, median(range)(n = 131)	27 (0–697)	17 (0–267)	
HIV viral level, median log copies/ml (range)(n = 57)	5.1 (2.6–5.9)	5.3 (1.7–5.9)	
Albumin, g/dl, median (range)	2.5 (0.6–3.7)	2.4 (1.0–3.8)	
LDH, U/l, median (range)	317 (129–851)	362 (119–962)	
Alveolar-arterial oxygen gradient, mm Hg, median (range)(n = 129)	41.5 (18.0–89.0)	43.4 (22.3–82.0)	

**Complications, n (%)**			
ICU admission	30 (23.6)	70 (42.4)	0.001
Mechanical ventilation	10 (7.9)	29 (17.6)	0.02
Pneumothorax	2 (1.6)	18 (10.9)	0.002
Respiratory failure after 5 days	1 (0.8)	9 (5.5)	NS
Change of PCP therapy	1 (0.8)	13 (7.9)	0.005

**HAART started in hospital, n (%)**	5 (7.7)	11 (10.5)	NS

**Outcome**			
Died in-hospital	8 (6.3)	26 (15.8)	0.01

Abbreviations: HAART, highly active antiretroviral therapy; ICU, intensive care unit; LDH, lactate dehydrogenase; NS, not significant; PCP, *Pneumocystis *pneumonia.*For overall comparison. p < 0.001 for blacks compared to Hispanic/Latinos and p = 0.018 for blacks compared to others/unknown. p-values adjusted for multiple comparisons.

### Predictors of mortality

Thirty-four patients (11.6%) died during hospitalization. ICU mortality was 29.0%. Univariate analyses to determine clinical variables associated with increased in-hospital mortality found that the need for mechanical ventilation (OR = 18.0, 95%CI = 7.9–41.1, p < 0.001), development of a pneumothorax (OR = 10.3, 95%CI = 3.9–27.3, p < 0.001), high serum lactate dehydrogenase (LDH)(OR = 1.3, 95%CI = 1.09–1.65, p = 0.005), and high alveolar-arterial oxygen gradient (OR = 1.08, 95%CI = 1.03–1.12, p = 0.001) predicted increased mortality (Table 3). High serum albumin (OR = 0.32, 95%CI = 0.17–0.60, p < 0.001) and an empiric diagnosis (OR = 0.36, 95%CI = 0.16–0.82, p = 0.02) were associated with decreased mortality. Other variables such as age or log-transformed age, history of PCP, CD4 cell count, HIV viral RNA levels, change in PCP treatment, and respiratory failure more than five days after hospital admission were not associated with mortality. Separate univariate analyses were performed for the definitive and empiric diagnoses groups and results were similar (Table 3). In addition, when empiric diagnosis was included in multivariate modeling of mortality adjusted for severity of illness, it did not have an independent association with mortality. Therefore, multivariate modeling was performed for all subjects and demonstrated that development of a pneumothorax (OR = 15.7, 95%CI = 4.4–56.4, p < 0.001) and need for mechanical ventilation (OR = 14.8 95%CI = 5.7–38.9, p < 0.001) were independent predictors of increased mortality (Table 3). Increasing serum albumin was independently associated with increased survival (OR = 0.27 per 0.1 g/dl increase, 95%CI = 0.12–0.61, p = 0.002). Alveolar-arterial oxygen gradient was available for only 129 subjects and was therefore not included in multivariate modeling as its inclusion decreased the power of the model.

**Table 3:** Predictors of in-hospital mortality for HIV-infected patients with *Pneumocystis *pneumonia for the entire cohort and according to empiric or definitive diagnosis.

**Univariate predictors**	**All subjects (n = 292) OR (95% CI), p-value**	**Empiric (n = 127) OR (95% CI), p-value**	**Definitive (n = 165) OR (95% CI), p-value**
Mechanical ventilation	18.0 (7.9–41.1), < 0.001	86.3 (13.1–568.3), <0.001	9.6 (3.8–24.7), < 0.001
Pneumothorax	10.3 (3.9–27.3), < 0.001	_*	10.2 (3.5–29.7), < 0.001
Serum LDH (per 100 U/l increase)	1.3 (1.09–1.65), 0.005	1.3 (0.79–2.02), 0.33	1.3 (1.02–1.63), 0.03
Serum albumin (per 0.1 g/dl increase)	0.32 (0.17–0.60), < 0.001	0.27 (0.09–0.88), 0.02	0.36 (0.17–0.77), 0.008
Empiric diagnosis	0.36 (0.16–0.82), 0.02	_	_
Alveolar-arterial oxygen gradient (per 1 mm Hg increase)(n = 129)	1.08 (1.03–1.12), 0.001	1.05 (0.98–1.12), p = 0.16	1.10 (1.03–1.17), p = 0.003

**Multivariate predictors (n = 278)**			
Pneumothorax	15.7 (4.4–56.4), < 0.001		
Mechanical ventilation	14.8 (5.7–38.9), < 0.001		
Serum albumin (per 0.1 g/dl increase)	0.27 (0.12–0.61), 0.002		

Abbreviations: CI, confidence interval; LDH, lactate dehydrogenase; OR, odds ratio.

*Unable to calculate odds ratio because of the small number of occurrences.

### Relationship of HAART and mortality

One hundred and seven patients (36.6%) received HAART prior to hospital admission. Duration of HAART use was available in 35 subjects (32.7%). Of those with a known duration, 51.4% had started HAART within one month of admission and 11.4% had started it within one to six months. Subjects receiving HAART prior to admission were similar to those not receiving HAART in age, gender, race/ethnicity, and HIV risk factor (Table 1). More subjects receiving HAART had a prior history of PCP (40.2% versus 14.6%, OR = 4.5, 95%CI = 2.50–7.97, p < 0.001) and were using PCP prophylaxis (56.1% vs. 16.9%, OR = 7.2, 95%CI = 4.10–12.53, p < 0.001). Thirty-six subjects (34.3%) reported that they were not taking their HAART regimens as prescribed. Laboratory values of CD4 cell count, plasma HIV viral levels, serum albumin, and LDH were comparable in the HAART and non-HAART groups. There was a trend for subjects on HAART to be admitted to the ICU less often (27.1% versus 37.1%, OR = 0.62, 95%CI = 0.37–1.05, p = 0.08) and to require mechanical ventilation less frequently (10.3% versus 14.0%, OR = 0.70, 95%CI = 0.33–1.49, p = 0.36) than the non-HAART subjects. Subjects on HAART were significantly less likely to develop a pneumothorax (1.9% vs. 9.6%, OR = 0.18, 95%CI = 0.041–0.80, p = 0.01). Length of stay was also shorter for those receiving HAART at admission (6 days vs. 9 days, p < 0.001).

Of those receiving HAART, 46 (43.0%) continued using HAART during admission. Sixteen patients (5.5%) started HAART during admission. HAART was started a median of 16 days after hospital admission (range 2–66) and a median of 9 days before discharge (range 2–28). Patients who continued or started HAART received it for a median of six days during admission (range 1–21). Approximately 15% of patients had at least one dose held during hospitalization. Reasons cited were the need to stop oral intake, development of side effects, or interaction with other medications.

Mortality was comparable for those on HAART at hospital admission and those not on HAART (9.3% vs. 11.8%, p = 0.52). Cox proportional survival modeling did not demonstrate any difference in survival to hospital discharge in those continued on HAART during hospitalization, those started on HAART while hospitalized, or these groups combined.

## Discussion and conclusion

This study demonstrated that in-hospital mortality of HIV-infected patients with PCP admitted to LAC-USC hospital from 2000 to 2003 was 11.6% and ICU mortality was 29.0%. The need for mechanical ventilation, development of a pneumothorax, and low serum albumin were found to be independent predictors of mortality. In contrast to our previous work [9], use of HAART during admission did not have an association with mortality.

As seen in previous studies of PCP, the majority of our patients were not using PCP prophylaxis. Although many should have been taking PCP prophylaxis based on clinical criteria, we cannot determine from chart review whether they were non-compliant with prescribed therapy, did not have therapy prescribed by their provider, or were not in care despite a known diagnosis of HIV infection. As also seen in previous studies, the admission with PCP was often the subject's first diagnosis of HIV infection. PCP diagnoses in HAART users may have represented failing HAART regimens or non-compliance with HAART. Immune reconstitution inflammatory syndrome (IRIS) might also have accounted for some of these diagnoses, although we were unable to distinguish these possibilities from chart review [16].

In our study, there were a large number of empirically-diagnosed patients who differed from those in whom definitive diagnosis was pursued. Those with an empiric diagnosis were more likely to have known HIV, a past history of PCP, and to be receiving HAART. Empiric diagnosis was associated with improved mortality, but this association did not persist after adjustment for severity of illness. Parada and colleagues also found that patients with an empiric diagnosis of PCP had comparable mortality to those with definitive diagnoses [17]. In contrast, several previous studies have found that empiric diagnosis is associated with worse mortality than definitive diagnosis. Bennett and colleagues reported that HIV-infected subjects who had empiric diagnoses of PCP had higher mortality when adjusted for severity of illness [18]. Miller reported that HIV-infected patients in the ICU with an empiric diagnosis of PCP had a mortality of 63% compared to 53% for definitive diagnosis, although this difference was not statistically significant [15]. In our institution, subjects who are more severely ill, particularly those who are intubated and in the ICU, are more likely to undergo bronchoscopy and have a microscopic diagnosis which likely explains the mortality differences. However, because we could not exclude the possibility that these patients did not in fact have PCP, we performed separate mortality analyses for definitive and empiric diagnostic groups and found similar predictors and therefore included both groups in our multivariate model. In addition, because many hospitals do not pursue definitive diagnosis, we felt that these results would reflect the general population of PCP patients.

Interestingly, empirically-diagnosed patients were much more likely to be black, which was the only characteristic independently associated with an empiric diagnosis. The reasons for this are unclear, but studies of the effects of race on invasive procedures in the non-HIV-infected population have found that blacks are less likely to undergo bronchoscopy when seriously ill, less likely to have cardiac catheterization when having an acute myocardial infarction, and less likely to have lung surgery when diagnosed with lung cancer [19-21]. Other studies have examined racial differences in PCP and HIV-related care. Bennett found that black and Hispanic patients with PCP were less likely to have a bronchoscopy and more likely to die, but this effect was actually a function of insurance and hospital characteristics [22]. Others have also found that HIV-infected patients are less likely to undergo bronchoscopy if they have Medicaid as opposed to private insurance [17]. We do not have insurance information on our patients to determine if it accounts for the racial difference in empiric diagnoses, but LAC-USC is a county hospital that typically serves patients who are underinsured or have no insurance. This finding suggests that there may still be some bias in performing procedures in certain groups of patients.

Due to the heterogeneity of patient populations, differing admission standards, influence of decisions to withdraw care, and variable clinical practices, it is difficult to compare our mortality results to previous studies. Reported hospital mortality of HIV-infected patients with PCP prior to HAART ranged from 13% to 25% [23,24]. In the early HAART era (1995–1997), PCP mortality was 11.3% [7]. We found a similar mortality of 11.6%, suggesting that overall mortality of PCP has not changed a great deal.

Mortality of PCP patients requiring intensive care ranged from 60 to 76% in the pre-HAART era, but improved somewhat in the early HAART era [9,25,26]. A previous study in San Francisco from 1996 through 2001 demonstrated a mortality of 55% among patients admitted to the ICU with PCP compared to 63% seen at the same institution during the years immediately before HAART [9,26]. Others have found mortality rates ranging from 53 to 62% in the early years of the HAART era [8,10].

Similar to another study which included the later HAART years [15], our study demonstrates that ICU mortality has continued to decrease with the current mortality at 29%. Increased use of non-invasive ventilation, more widespread use of low tidal volume ventilation, and restriction of intravenous fluids might have improved mortality as there have been no significant changes in care of PCP to account for differences. Our cohort might have been healthier than those in previous studies, but most patients had advanced AIDS with low CD4 cell counts. ICU admission standards might also affect mortality. Our ICU has no set guidelines for ICU admission and transfer and initial admission to the ICU is at the discretion of the admitting physician. In fact, a higher percentage of our PCP patients (34%) were admitted to the ICU compared to only 16% in the study by Miller and colleagues [15]. Despite the difference in admission rates, our ICU mortality (29%) was quite similar to theirs (34%).

The predictors of mortality that we identified were consistent with previous studies. Mechanical ventilation, development of a pneumothorax, and low serum albumin were associated with a poor outcome. These factors have all been previously found to predict outcome in patients with PCP or in those with HIV infection [9,11,13,14,27]. In contrast to some previous studies, ICU admission after several days of treatment was not associated with increased mortality [9,13,15,28].

One potential factor that could influence current mortality is the use of HAART. A previous study demonstrated that PCP patients who received HAART during their ICU admission had a mortality rate of only 25% compared to 63% for those who did not [9]. In contrast, Miller reported low ICU mortality despite the fact that no patients were receiving HAART before or during admission. These results suggest that improvement in the HAART era was the result of improvements in ICU care, but there was no comparison group receiving HAART. Two other studies have not found a difference in ICU mortality with and without HAART, but these studies have not examined those with PCP directly [10,29]. There have been no prior studies examining HAART in non-ICU patients with PCP.

The current study adds to previous work by examining a larger group of patients, by including both patients with and without HAART use as well as those not requiring ICU care, and by performing detailed analyses of HAART use. Overall, there was no significant relationship between HAART and survival. We found no mortality effect in patients who started HAART or who had it continued in the hospital. Our subjects started HAART, on average, more than two weeks after hospital admission and received it only for an average of six days, which likely would not be long enough to see a beneficial effect. Patients in the previous study reporting a beneficial effect of HAART might have received it for a longer time or more quickly after hospital admission than the current patients, but these data were not collected [9]. Other explanations of the inconsistent results are differences in patient populations or that improvements in ICU care of PCP patients have had a greater impact on mortality than the effects of HAART. There are significant risks associated with initiating HAART in the acute setting including difficulties with administration and absorption that could lead to resistance, drug toxicities and interactions, and the development of IRIS. Given the low numbers of subjects receiving HAART in this study and the many factors that can affect both the clinical decision and the ability to start HAART in the hospital, our results need to be interpreted with caution, but they do not suggest a benefit from HAART in this setting.

Another area of controversy has been whether patients receiving HAART present differently from those not receiving HAART at admission. Although HAART patients generally had similar serum albumin and LDH levels, they were less likely to have a pneumothorax and tended to be less likely to require mechanical ventilation or ICU admission and thus seemed to have had less severe illness. This finding might be due to a tendency for subjects who were in care and with known HIV status to seek medical attention at an earlier point in the disease or there might be some differences in the presentation of the disease in HAART users. In addition, HAART use prior to admission was associated with a shorter length of stay, possibly reflecting more stable housing situations or perhaps indicating a beneficial effect of HAART separate from any effects on mortality. Patients who reported using HAART at admission did not actually have lower HIV viral RNA levels than those not on HAART. There are several potential explanations of the detectable viral levels in these patients. First, they may not have been compliant with the regimens as approximately one-third of subjects reported not taking their medications as prescribed. Also, of the subjects in whom length of HAART use was known, many had recently started HAART and might not have had sufficient time to see benefit. Finally, HAART might not have been effective in these selected patients as those with successful HAART use would not be expected to develop PCP.

There are several limitations of this study. First, it is retrospective and from a single center. Different populations or different hospitals could be expected to have different results. Factors such as criteria for ICU admission and views of limitations of care could influence outcomes. Another difficulty is that our analysis of the effects of HAART on mortality might be altered by the fact that patients selected to start HAART either before or during admission may differ from those not interested in or not offered the therapy. Also, pre-hospital data regarding markers of HIV infection and prognosis were not available for a number of subjects, limiting our ability to analyze the effect of these factors on in-hospital outcome. Finally, we might have lacked sufficient power to detect a difference in subjects on HAART. Larger, prospective studies would be needed to determine the effects of initiating HAART during hospitalization for acute PCP.

In summary, overall hospital mortality for PCP is similar to that reported earlier in the HAART era, but ICU mortality appears to be lower than that previously reported at other centers. Whether current ICU mortality represents improvements in general ICU care or changes in the HIV-infected population is unknown, but should provide clinicians with justification and optimism for continued ICU care of these patients. Predictors of mortality have not changed in the recent HAART era and need for mechanical ventilation, development of a pneumothorax, and low serum albumin still portend a poor outcome. PCP diagnoses in HAART users likely represented failing HAART regimens or non-compliance with HAART. Administration of HAART during hospitalization or continuation of a potentially failing HAART regimen was not associated with a decrease in mortality, but larger, prospective studies are needed to confirm the true relationship of HAART to outcome of PCP.

## Abbreviations

AIDS: acquired immunodeficiency syndrome; HAART: highly active antiretroviral therapy; HIV: human immunodeficiency virus; ICU: intensive care unit; IRIS: immune reconstitution inflammatory syndrome; LAC-USC: Los Angeles County-University of Southern California; LDH: lactate dehydrogenase; PCP: *Pneumocystis *pneumonia

## Competing interests

The authors declare that they have no competing interests.

## Authors' contributions

SR carried out data acquisition, performed statistical analyses, and drafted the manuscript.

TA participated in data acquisition.

MU participated in data acquisition.

SS participated in data acquisition.

AM conceived of the study, participated in its design and coordination, and revised the manuscript.

All authors read and approved the final manuscript.

## Pre-publication history

The pre-publication history for this paper can be accessed here:



## References

[B1] Dore GJ, Li Y, McDonald A, Ree H, Kaldo JM (2002). Impact of highly active antiretroviral therapy on individual AIDS-defining illness incidence and survival in Australia. J Acquir Immune Defic Syndr.

[B2] Palella FJ, Delaney KM, Moorman AC, Loveless MO, Fuhrer J, Satten GA, Aschman DJ, Holmberg SD (1998). Declining morbidity and mortality among patients with advanced human immunodeficiency virus infection. HIV Outpatient Study Investigators. N Engl J Med.

[B3] Kaplan JE, Hanson D, Dworkin MS, Frederick T, Bertolli J, Lindegren ML, Holmberg S, Jones JL (2000). Epidemiology of human immunodeficiency virus-associated opportunistic infections in the United States in the era of highly active antiretroviral therapy. Clin Infect Dis.

[B4] Mocroft A, Katlama C, Johnson AM, Pradier C, Antunes F, Mulcahy F, Chiesi A, Phillips AN, Kirk O, Lundgren JD (2000). AIDS across Europe, 1994–98: the EuroSIDA study. Lancet.

[B5] Detels R, Tarwater P, Phair JP, Margolick J, Riddler SA, Munoz A (2001). Effectiveness of potent antiretroviral therapies on the incidence of opportunistic infections before and after AIDS diagnosis. AIDS.

[B6] Morris A, Lundgren JD, Masur H, Walzer PD, Hanson DL, Frederick T, Huang L, Beard CB, Kaplan JE (2004). Current epidemiology of *Pneumocystis *pneumonia. Emerg Infect Dis.

[B7] Arozullah AM, Yarnold PR, Weinstein RA, Nwadiaro N, McIlraith TB, Chmiel JS, Sipler AM, Chan C, Goetz MB, Schwartz DN (2000). A new preadmission staging system for predicting inpatient mortality from HIV-associated *Pneumocystis carinii *pneumonia in the early highly active antiretroviral therapy (HAART) era. Am J Respir Crit Care Med.

[B8] Curtis RJ, Yarnold PR, Schwartz DN, Weinstein RA, Bennett CL (2000). Improvements in outcomes of acute respiratory failure for patients with human immunodeficiency virus-related *Pneumocystis carinii *pneumonia. Am J Respir Crit Care Med.

[B9] Morris A, Wachter RM, Luce J, Turner J, Huang L (2003). Improved survival with highly active antiretroviral therapy in HIV-infected patients with severe *Pneumocystis carinii *pneumonia. AIDS.

[B10] Khouli H, Afrasiabi A, Shibli M, Hajal R, Barrett CR, Homel P (2005). Outcome of critically ill human immunodeficiency virus-infected patients in the era of highly active antiretroviral therapy. J Intensive Care Med.

[B11] Afessa B, Green B (2000). Clinical course, prognostic factors, and outcome prediction for HIV patients in the ICU. The PIP (Pulmonary complications, ICU support, and prognostic factors in hospitalized patients with HIV) study. Chest.

[B12] Alves C, Nicolas JM, Miro JM, Torres A, Agusti C, Gonzalez J, Rano A, Benito N, Moreno A, Garcia F (2001). Reappraisal of the aetiology and prognostic factors of severe acute respiratory failure in HIV patients. Eur Respir J.

[B13] Bedos JP, Dumoulin JL, Gachot B, Veber B, Wolff M, Regnier B, Chevret S (1999). *Pneumocystis carinii *pneumonia requiring intensive care management: survival and prognostic study in 110 patients with human immunodeficiency virus. Crit Care Med.

[B14] De Palo VA, Millstein BH, Mayo PH, Salzman SH, Rosen MJ (1995). Outcome of intensive care in patients with HIV infection. Chest.

[B15] Miller RF, Allen E, Copas A, Singer M, Edwards SG (2006). Improved survival for HIV infected patients with severe *Pneumocystis jirovecii *pneumonia is independent of highly active antiretroviral therapy. Thorax.

[B16] Barry SM, Lipman MC, Deery AR, Johnson MA, Janossy G (2002). Immune reconstitution pneumonitis following *Pneumocystis carinii *pneumonia in HIV-infected subjects. HIV Med.

[B17] Parada JP, Deloria-Knoll M, Chmiel JS, Arozullah AM, Phan L, Ali SN, Goetz MB, Weinstein RA, Campo R, Jacobson J (2003). Relationship between health insurance and medical care for patients hospitalized with human immunodeficiency virus-related *Pneumocystis carinii *pneumonia, 1995–1997: Medicaid, bronchoscopy, and survival. Clin Infect Dis.

[B18] Bennett CL, Horner RD, Weinstein RA, Kessler HA, Dickinson GM, Pitrak DL, Gilman SC, George WL, Cohn SE, Simberkoff MS (1995). Empirically treated *Pneumocystis carinii *pneumonia in Los Angeles, Chicago, and Miami: 1987–1990. J Infect Dis.

[B19] Phillips RS, Hamel MB, Teno JM, Bellamy P, Broste SK, Califf RM, Vidaillet H, Davis RB, Muhlbaier LH, Connors AF (1996). Race, resource use, and survival in seriously ill hospitalized adults. The SUPPORT Investigators. J Gen Intern Med.

[B20] Bach PB, Cramer LD, Warren JL, Begg CB (1999). Racial differences in the treatment of early-stage lung cancer. N Engl J Med.

[B21] Bertoni AG, Goonan KL, Bonds DE, Whitt MC, Goff DC, Brancati FL (2005). Racial and ethnic disparities in cardiac catheterization for acute myocardial infarction in the United States, 1995–2001. J Natl Med Assoc.

[B22] Bennett CL, Horner RD, Weinstein RA, Dickinson GM, DeHovitz JA, Cohn SE, Kessler HA, Jacobson J, Goetz MB, Simberkoff M (1995). Racial differences in care among hospitalized patients with *Pneumocystis carinii *pneumonia in Chicago, New York, Los Angeles, Miami, and Raleigh-Durham. Arch Int Med.

[B23] Curtis JR, Greenberg DL, Hudson LD, Fisher LD, Krone MR, Collier AC (1994). Changing use of intensive care for HIV-infected patients with *Pneumocystis carinii *pneumonia. Am J Respir Crit Care Med.

[B24] Mansharamani NG, Garland R, Delaney D, Koziel H (2000). Management and outcome patterns for adult *Pneumocystis carinii *pneumonia, 1985 to 1995: comparison of HIV-associated cases to other immunocompromised states. Chest.

[B25] Wachter RM, Russi MB, Bloch DA, Hopewell PC, Luce JM (1991). *Pneumocystis carinii *pneumonia and respiratory failure in AIDS. Improved outcomes and increased use of intensive care units. Am Rev Respir Dis.

[B26] Nickas G, Wachter RM (2000). Outcomes of intensive care for patients with human immunodeficiency virus infection. Arch Intern Med.

[B27] Gill JK, Greene L, Miller R, Pozniak A, Cartledge J, Fisher M, Nelson MR, Soni N (1999). ICU admission in patients infected with the human immunodeficiency virus – a multicentre survey. Anaesthesia.

[B28] Forrest DM, Zala C, Djurdjev O, Singer J, Craib KJ, Lawson L, Russell JA, Montaner JS (1999). Determinants of short- and long-term outcome in patients with respiratory failure caused by AIDS-related *Pneumocystis carinii *pneumonia. Arch Intern Med.

[B29] Casalino E, Wolff M, Ravaud P, Choquet C, Bruneel F, Regnier B (2004). Impact of HAART advent on admission patterns and survival in HIV-infected patients admitted to an intensive care unit. AIDS.

